# Fluoroless intravascular ultrasound image-guided liver navigation in porcine models

**DOI:** 10.1186/s12876-021-01600-3

**Published:** 2021-01-09

**Authors:** Takeshi Urade, Juan Manuel Verde, Alain García Vázquez, Konstanze Gunzert, Patrick Pessaux, Jacques Marescaux, Mariano Eduardo Giménez

**Affiliations:** 1 Institute of Image-Guided Surgery, IHU Strasbourg, 1, place de l’Hôpital, 67091 Strasbourg Cedex, France; 2 IRCAD, Research Institute Against Digestive Cancer, Strasbourg, France; 3 Siemens Healthcare SAS, Strasbourg, France; 4 Department of General, Digestive and Endocrine Surgery, Nouvel Hôpital Civil, University of Strasbourg, Strasbourg, France

**Keywords:** Fluoroless, Image guidance, Intravascular, Liver, Navigation, Ultrasound catheter

## Abstract

**Background:**

An intravascular ultrasound catheter (IVUSc) was developed for intracardiac ultrasound to assess interventions with compelling results. However, intrahepatic vascular exploration was rarely tested and was always associated with X-ray techniques. The aim of this study was to demonstrate the feasibility to navigate through the whole liver using an IVUSc, providing high-quality images and making it unnecessary to use ionizing radiation.

**Methods:**

An ex vivo pig visceral block and an in vivo pig model were used in this study. The IVUS equipment was composed of an US system, and of an 8 French lateral firing IVUSc capable of producing 90-degree sector images in the longitudinal plane. After accessing the intravascular space with the IVUSc into the models, predetermined anatomical landmarks were visualized from the inferior vena cava and hepatic veins and corroborated.

**Results:**

IVUS navigation was achieved in both models successfully. The entire navigation protocol took 87 and 48 min respectively, and 100% (21/21) and 96.15% (25/26) of the landmarks were correctly identified with the IVUSc alone in the ex vivo and in vivo models respectively. IVUS allowed to clearly visualize the vasculature beyond third-order branches of the hepatic and portal veins.

**Conclusions:**

A complete IVUS liver navigation is feasible using the IVUSc alone, making it unnecessary to use ionizing radiation. This approach provides high-definition and real-time images of the complex liver structure and offers a great potential for future clinical applications during diagnostic and therapeutic interventions.

## Background

A wide range of image-guided strategies and techniques have been used to approach diseased livers with diagnostic, theragnostic, or therapeutic intentions, with the aim to decrease the invasiveness and the rate of complications. The liver motion and deformation, as well as its complex anatomy comprising major vascular and biliary structures, constantly challenge the implementation of image-guided minimally invasive techniques. From the hepatic imaging repertoire, ultrasound (US) excels the others because it is innocuous (non-ionizing) and it provides a real-time assessment of the target and/or the environment, hence increasing situational awareness and spatial orientation. As it addresses the abovementioned issues, US is nowadays broadly integrated in almost all interventional and surgical workflows as a first-line tool, exposing its limitations and drawbacks. The depth/distance-associated low resolution, along with air-related interference in the course of percutaneous interventions, the high number of time-consuming iterations required during liver surgery and ablations, as well as its long learning curves, confirm the foremost drawbacks of US.

In an attempt to address these issues, other disciplines started to use intravascular ultrasound catheters (IVUSc) to assess the procedures with image guidance, reporting promising results in the literature, being widely adopted in the end, hence expanding their cardiac indications. An IVUSc was developed for intracardiac and intraluminal exploration, and during cardiovascular interventions, intracardiac echocardiography was reported as a powerful guide in multiple procedures (atrial defects and patent foramen ovale closures [1, 2], and ablation [3, 4]. These IVUS approaches were scarcely attempted in the liver, and as far as we know, they were exclusively related to transjugular intrahepatic portosystemic shunts (TIPS) [5–9] and direct intrahepatic portocaval shunts (DIPS) [10–12], using ionizing radiation and remaining in the lumen of the inferior vena cava (IVC). To our knowledge, there is only one report describing intrahepatic IVUS navigation through hepatic veins with diagnostic intentions, but also relying on fluoroscopic guidance [13].

We envision several potential indications of the IVUSc in the diagnostic, theragnostic and therapeutic liver fields. Accordingly, we created and developed a dual ex vivo and in vivo test aiming to explore the feasibility of navigating the liver beyond the IVC and through the hepatic veins (HVs), without using X-rays.

## Methods

The study was performed in strict accordance with the recommendations available in the Guide for the Care and Use of Laboratory Animals of the National Institutes of Health [14], after obtaining full approval from the Institutional and French National Ethics Committee (APAFIS #16886-2018092712542067v2), and in full compliance with French laws for animal use and care, and also with the directives of the European Community Council (2010/63/EU). The written consent was not needed because this study included only animal models. All sections of this report adhere to the ARRIVE Guidelines for reporting animal research [15]. This experimental study was designed in compliance with the 3Rs principles (Replacement, Refinement, and Reduction), and developed in congruence with the best welfare animal conditions [16]. Accordingly, it started ex vivo as a proof-of-concept and was performed in one pig model aiming to replicate human characters, facilitating its reproducibility and potential transfer. One 56 kg male pig (*Sus Scrofa Domesticus, ssp.* Large White; Comptoir Agricole, France), and one thoraco-abdominal pig visceral block were used. The pig was housed in a group, and acclimatized for 48 h in an enriched environment, respecting circadian cycles of light-darkness, and with constant humidity and temperature conditions. It was fasted 24 h before surgery, with ad libitum access to water, and finally sedated (zolazepam + tiletamine 10 mg/kg IM) 30 min before the procedure to decrease stress. Anesthesia induction was achieved by means of intravenous (18 G IV catheter in ear vein) propofol 3 mg/kg, and maintained with rocuronium 0.8 mg/kg along with inhaled isoflurane 2%. At the end, the animal was euthanized with an IV lethal dose of pentobarbital (40 mg/kg) and checked twice for vital signs before it was properly discarded. The study was conducted in the animal dedicated hybrid operating room of the IHU Strasbourg and the animal facilities of the Research Institute against Digestive Cancer (IRCAD), in Strasbourg, France.

For imaging purposes, two US systems were used, one with an intravascular transducer and the other with an extracorporeal transducer. The intravascular US system (ACUSON S3000 HELX Touch Ultrasound System, Siemens Healthineers, Germany), equipped with a cardiac package, a special connector (ACUSON Swift-Link™), and an 8 French lateral firing IVUS catheter (ACUSON AcuNav™, 4–10 MHz) with a four-way steering in two planes, each direction via 160 degrees, producing images in the longitudinal plane and providing color Doppler tissue imaging capabilities on demand. The second system, called external system, consisted of an additional US system (ACUSON S3000 Ultrasound System), with a convex transducer (6C1HD, 1–6 MHz).

**Fig. 1:**
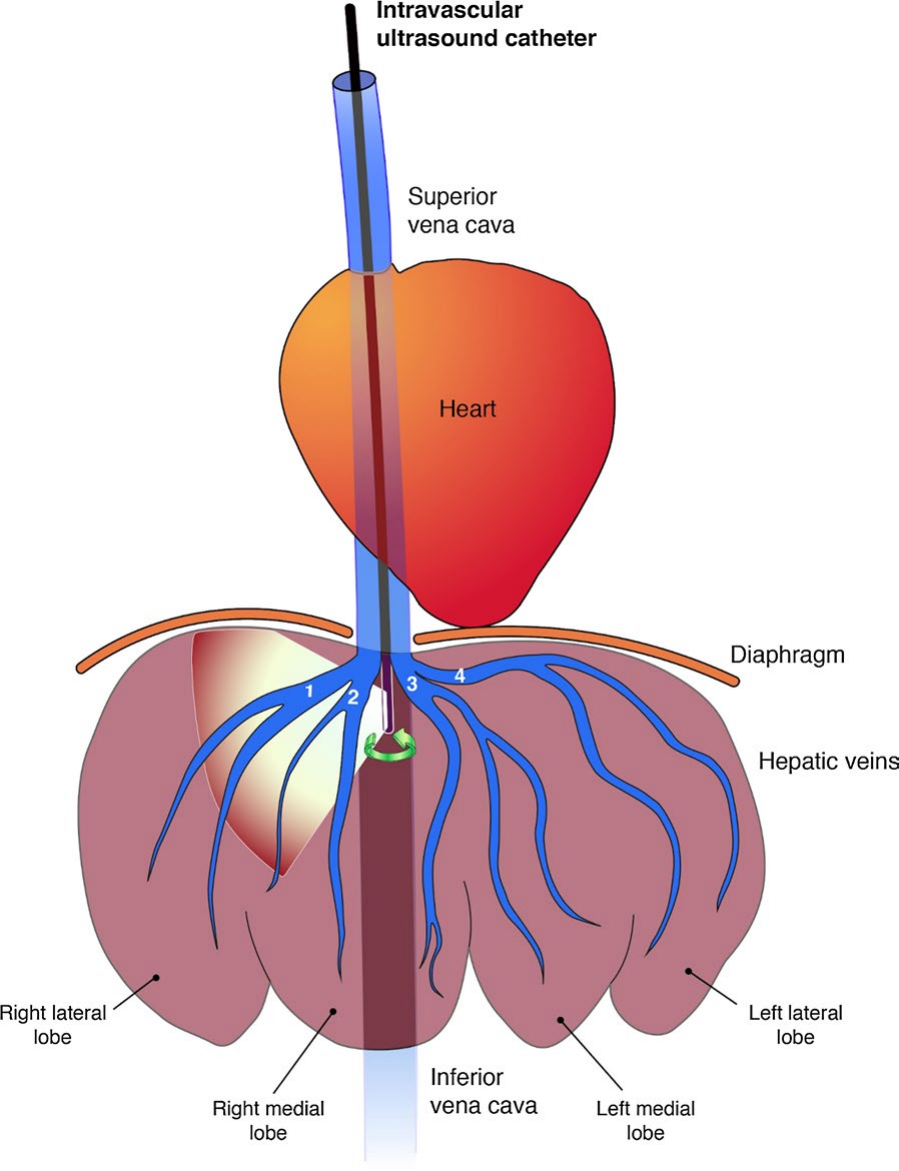
Intravascular ultrasound image-guided liver navigation. 1, right hepatic vein; 2, right median hepatic vein; 3, left median hepatic vein; 4, left lateral hepatic vein. The hepatic veins were classified according to the information given by Nykonenko et al. [24]. The illustration was originally created by the authors

For the ex vivo model, a pig visceral block (comprising heart, IVC, liver) was partially mounted in a gelatin-based medium (200 bloom gelatin powder, Louis François, France—fast technique) inside a large plastic container. Both the portal vein (PV) (18 French Silicone Tiemann Foley Catheter, Teleflex Medical, USA), and the common bile duct (cholangiography catheter) were perfused with a warm (60℃) saline solution. A 9 French vascular introducer (Radiofocus, TERUMO Corp., Japan) was placed through the right atrium in the IVC, and the model was covered with a saline solution to improve US visualization, preventing air interference as well as organ motion/deformation related to probe-tissue interaction. After initiating the experiment, a regular intravascular flow/pressure (PV to HVs) circulation was achieved using a tripod to suspend the saline solution container at a height of 2 m. The bile duct catheter was closed but ready to be used according to the needs. For the in vivo pig model, the same abovementioned 9 French vascular introducer was introduced under US guidance into the right jugular vein (RJV) to grant the access of the IVUSc (Fig. 1).

The success of the US-guided intravascular navigation of the liver using the IVUSc was defined beforehand as the precise recognition of more than 95% of the prearranged anatomical landmarks, only relying upon the images provided by the IVUSc without any further complementary methods. The landmarks were selected cephalocaudally, starting immediately after passing through a central vein, and ending with extrahepatic neighboring structures, namely thoracic IVC, retrohepatic IVC, infrahepatic IVC, HVs confluence, right HV, right median HV, left median HV, left lateral HV, third-order HV branches, main PV, right PV, left PV, third-order PV branches, common bile duct, right hepatic bile duct, left hepatic bile duct, gallbladder with the access to four HVs in an ex vivo model, and the abovementioned structures with hepatic artery, right hepatic artery, left hepatic artery, pancreas, and duodenum in an in vivo model (see Additional file 1: Supplementary file 1 and Additional file 2: Supplementary file 2, which illustrate the data collection sheets for IVUS image-guided liver navigation). Both ex vivo/in vivo tests started when operator #1 introduced the IVUSc through the vascular introducer into a central vein and navigated the liver stepwise with the IVUS information alone. The partial times elapsed to identify each landmark were annotated, and when each of them was identified by operator #1, two other experienced US operators (#2 and #3) checked the indicated location with the secondary US system using a convex external probe and collected them as categorical variables (yes/no—checkboxes), along with the timestamp and video images of both US systems. No feedback was given back to operator #1 to avoid intraprocedure external biases. As a contingency plan, the experiment was scheduled in the hybrid operating room, and two extra scenarios were prepared in case of failure of the IVUS navigation alone: one making the secondary external US system available to operator #1 (second scenario), and the other incorporating X-ray fluoroscopy and/or cone-beam computed tomography images (Artis Zeego, Siemens Healthineers).

## Results

All IVUS scans were performed in B and/or color Doppler mode, using a 7 MHz frequency with a mechanical index ranging from 0.8 to 1.0, and the gain and focus were adapted to visualize landmarks properly. The resulting image was inverted both up/down and right/left to display the correct anatomical orientation during liver navigation.

In the ex vivo settings, the entire navigation protocol took 87 min, and all of the 21 (100%) preselected landmarks were recognized. In the in vivo pig model, IVUS navigation was accomplished without the need for any other extra medical imaging method (US/X-rays), recognizing 96.15% (25/26, left lateral HV recognized later, see below) of landmarks in a total time of 48 min.

All expectations were achieved using the ex vivo model and it is worth mentioning that intravascular perfusion, through the PV to the HVs with a saline solution, allowed the correct configuration of both venous inflow and outflow as well as a positive Doppler signal helping to recognize vascular structures. Similarly, the underwater submersion of the model helped to avoid air-related interferences as well as unnecessary motion and deformations related to probe-tissue interaction. Under such circumstances, the hepatic artery (HA) could not be recognized, its lack of perfusion and circulation meant that it was impossible to use Doppler/color modes to assist with identification.

**Fig. 2:**
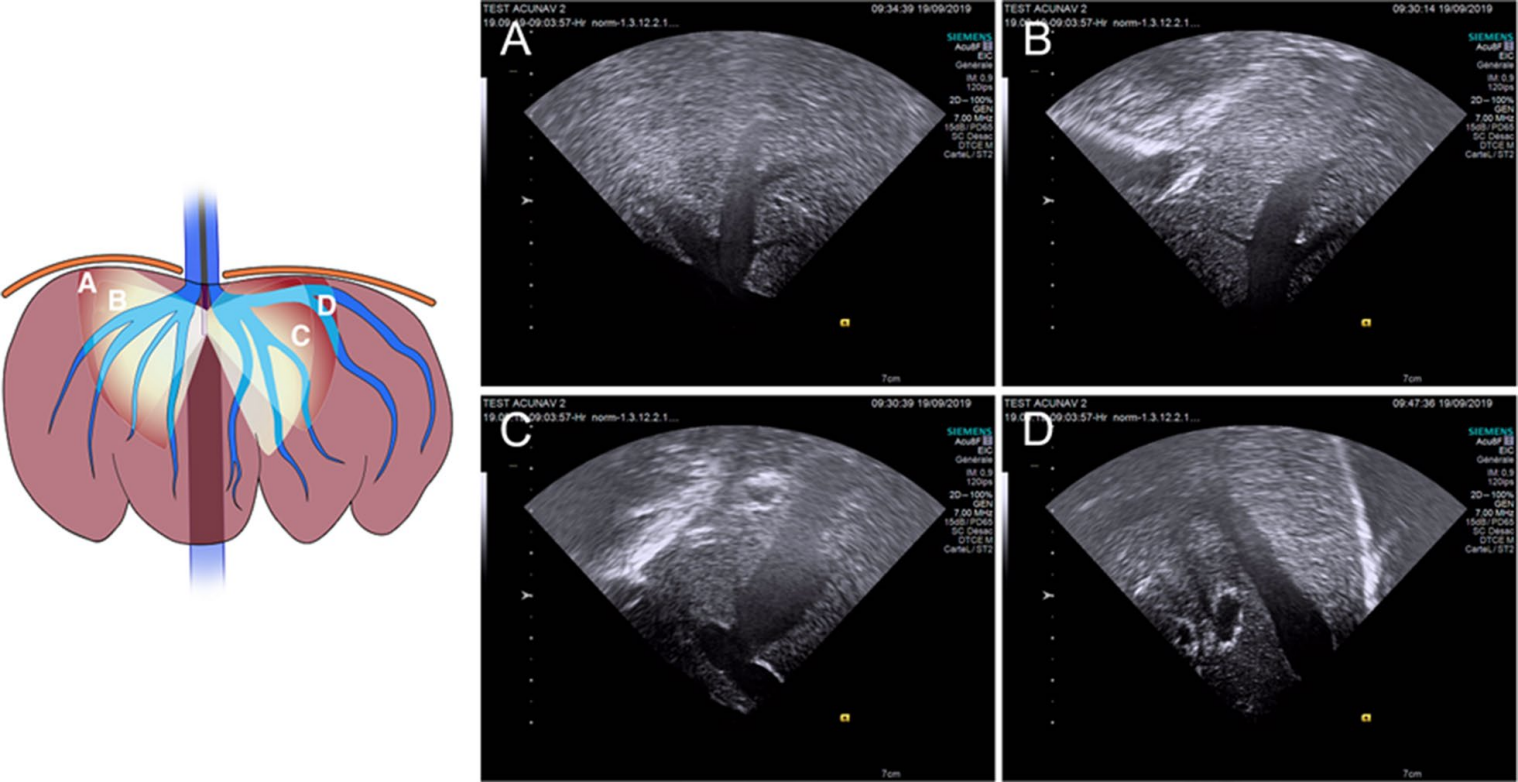
Intravascular ultrasound images of the hepatic veins visualized from the inferior vena cava. The schematic includes four planes of the ultrasound images corresponding to A, B, C, and D. The right side of the image is proximal and the left one is distal. **A** Right hepatic vein, **B** Right median hepatic vein, **C** Left median hepatic vein, **D** Left lateral hepatic vein. The illustration was originally created by the authors

**Fig. 3:**
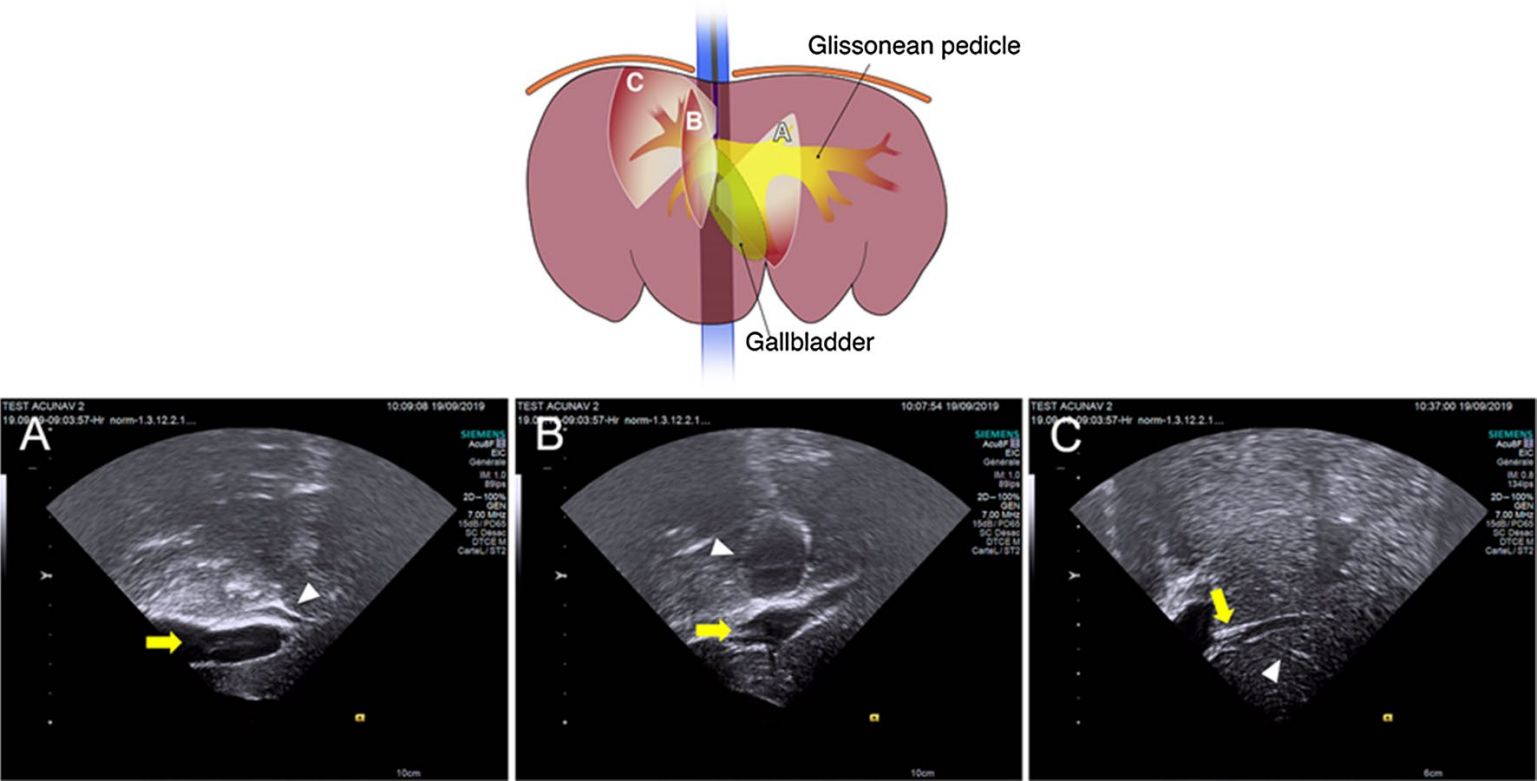
Intravascular ultrasound images of the portal veins and biliary system from the inferior vena cava. The schematic includes three planes of the ultrasound images corresponding to A, B, and C. The right side of the image is proximal and the left one is distal. **A** Main portal vein (yellow arrow) and right hepatic duct (white arrowhead), **B** Right portal vein (yellow arrow) and gallbladder (white arrowhead), **C** Third and fourth order of the portal vein (yellow arrow and white arrowhead). The illustration was originally created by the authors

In the in vivo settings, the RJV approach was the one which best matched the IVUSc length. However, the left side was also tested without any further difficulties or complications. The IVC navigation did not present any difficulties, and the HV outlets could be recognized via mere rotations of the IVUSc (Fig. 2). At this point, the IVUS operator misrecognized the left lateral HV and the left median HV, interpreting that they converged into a common trunk in the IVC, and this issue was later corrected during their cannulation maneuvers. The catheter had to be taken out twice after the concomitance of imaging artifacts and a loss of control of the endoscope’s handle (latterly attributed to the bending of the catheter tip, which went unnoticed at the time), even with the control wheels in the neutral position. The PV, the right and left trunks, and also third- and fourth-order branches (same for the HVs) were individually visualized as well as both main branches of the common bile duct (Fig. 3), while the HA and its right and left branches required Doppler/color modes to be adequately detected and recognized.

## Discussion

This study showed that IVUS liver navigation using a catheter proved to be a powerful tool to explore the liver without exposure to radiation (or the need for other medical imaging systems). The image quality of the liver parenchyma, vascular structures and biliary elements, as well as intravascular navigation, exceeded expectations, inspiring researchers to envision several potential indications in the liver and also beyond it.

This approach has several strengths and advantages. The first advantage is related to the 4 degrees of freedom (pitch, roll, yaw, and translation), its steering properties and real-time images, facilitating intrahepatic navigation. As a result, high-quality images beyond third-order vascular structures (PV and HV) can be acquired from the IVC, but also from the interior of any cannulated HV. Peripheral and central branches, usually inconspicuous due to their deep location inside the liver, can be observed as well. In addition, the intravascular space avoids gas-related interferences and liver deformations, contributing to a secondary point of view, which can work synergically with other medical imaging techniques. Doppler/color modes were essential tools during the entire experiment, and mandatory to deal with the HA and its branches. Similarly, other US imaging modalities such as shear wave elastography, contrast-enhanced ultrasound and image fusion which are currently unavailable for the abovementioned IVUSc, could well enhance this approach and extend indications even further.

**Fig. 4:**
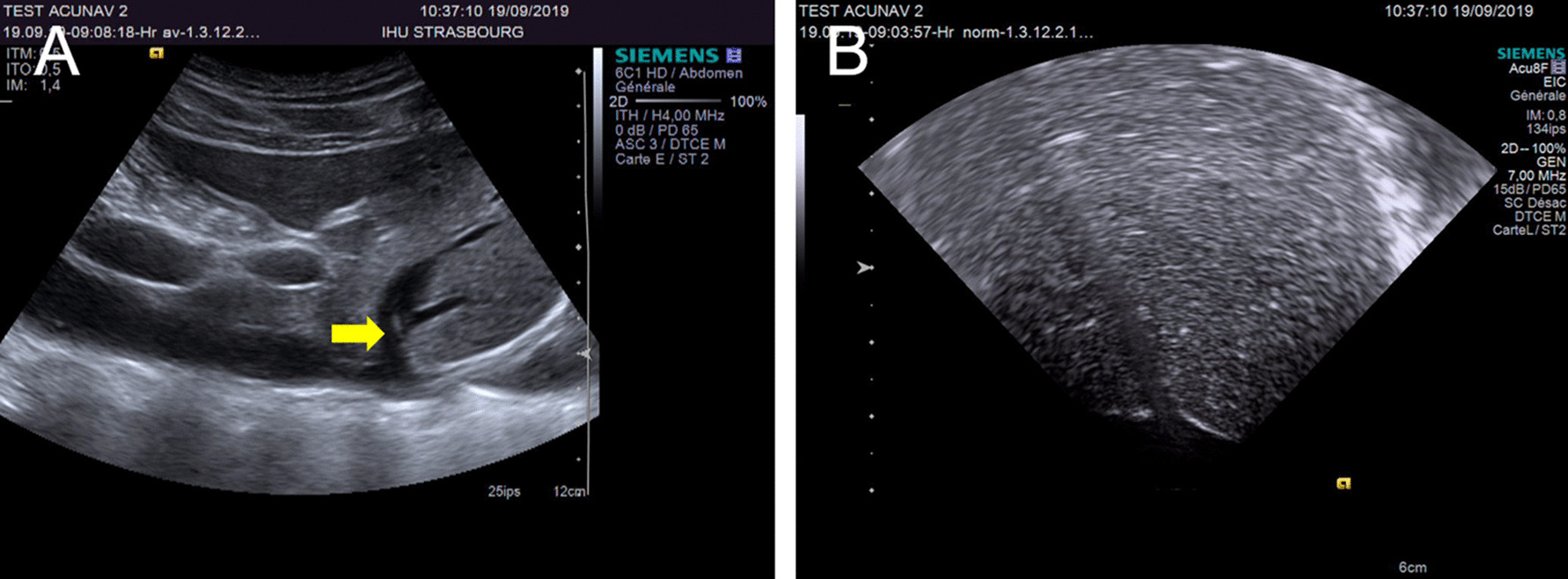
External ultrasound enabled confirmation of the location of the intravascular catheter. **a** External ultrasound detected the intravascular catheter tip (yellow arrow) in the left median hepatic vein. The right side of the image is cranial and the left one is caudal. **b** Intravascular ultrasound image from the left median hepatic vein. The right side of the image is proximal and the left one is distal

A second and considerable advantage is the lack of exposure to ionizing radiation of the patient and the team, the harmful effect of which has been extensively demonstrated among physicians performing interventional procedures. It is reported to be associated with an increased incidence of lens opacities and left-sided brain tumors [17–19]. Gottardi et al. [13] reported the need for extra doses of ionizing radiation to guide and verify IVUSc positions, and major limitations to access the left hepatic vein resulting in incomplete evaluations. Although we used porcine models, we managed to navigate the entire liver without radiation, and it is also worth mentioning that the combination of IVUS and other US systems (percutaneous, laparoscopic, etc.) should ideally be considered as an alternative to X-ray, or at least be implemented as a first step before the use of X-ray-based techniques, in order to potentially decrease the radiation dose (Fig. 4).

Among a wide list of potential translations of this approach to other organs, we can envisage multiple indications in the liver and beyond it, using the jugular approach and also femoral vessels (maybe also avoiding general anesthesia). US is commonly used for the intraoperative detection and location of liver lesions, and as a guidance to demarcate and assess liver transections (open, laparoscopic, and robotic). However, this extrahepatic US guidance cannot be performed simultaneously, requires multiple instrument iterations, and is more demanding, extending surgical time. With the IVUSc, both problems can be addressed simultaneously, planning the transection line, its demarcation, and real-time instrumental visualization. As Abdelaziz et al. [20] described in the perioperative liver transplantation phase, ultrasound represents a powerful tool which can be used on demand and in real time to assess the patency and/or potential complications of vascular anastomoses. Considering that these patients usually have a central line during this period, we might envision a role for IVUS, avoiding the use of transesophageal echocardiography as reported by Khurmi et al. [21].

In the field of image-guided minimally invasive procedures, this IVUS approach can be conceived as a guidance system, allowing and enhancing the guidance, navigation, and control modalities of needle-based procedures, relying upon two real-time US systems (external and intravascular), with two different and synergic points of view. Even more, in the dynamic and growing field of liver ablation, some weaknesses of this technique in terms of precision and accuracy, as well as the real-time control of the results (“shadowing effect”), can be potentially mitigated. With this approach, we can expect to considerably decrease the X-ray dose (and maybe avoid it completely), as well as decrease contrast medium volumes, providing better images than the reported use of IVUS during TIPS/DIPS [6–12], always relying on X-rays and placing the catheter in the interior of the IVC. In addition, this hepatoportal communication can be used to access the PV with IVUS navigation, seeking diagnostic or interventional intentions. Extending previous works such as the one of Kaneko et al. [22] describing the role of the intraportal endovascular ultrasound assessment to determine portal venous invasion, and the report of Chick et al. [23] using IVUS guidance to perform a transbiliary biopsy of pancreatobiliary carcinomas, further potential indications can be conceived beyond the liver and related to the exploration of abdominal regions which are traditionally difficult to approach with US (e.g. pancreatic necrosis, borderline resectable pancreatic cancer, or retroperitoneal hematoma, etc.), or even outside blood vessels (e.g. biliary tree exploration).

This study has the limitations of a proof-of-concept translational study, and it should be tested and evaluated exhaustively before being used in humans. The abovementioned technique is demanding (HV cannulation/US planes recognition), and will potentially have steep learning curves, even in professionals with US skills. At this point, it is worth mentioning that ex vivo models such as the one developed for this study, are good enough to acquire these skills and abilities before moving on to living tissues. The lack of contrast enhancement, shear-wave elastography, and image fusion capabilities put this approach at a disadvantage as compared to other US assessments, so their incorporation might be important. The damage to blood vessels (thermal/mechanical) when in direct contact with them should be meticulously studied. Concerning the used catheter, when it is manipulated as a vascular catheter without control of the steering knobs, the tip can be bent and the steering knobs remain in a neutral position, thereby producing an inversion of the handle system (e.g. right–left mirror images).

## Conclusions

This study clearly shows that it is feasible to complete the entire navigation of the liver through the IVC and HV only relying upon IVUS images, without the need for any further X-ray imaging techniques. This approach provides a high-definition and real-time visualization of vascular structures beyond third-order branches, as well as those located in central positions. This approach has great potential for many clinical applications in both diagnostic and therapeutic fields. As a result, further investigations are required to ensure a secure and profitable translation to humans.

## Supplementary Information

**Additional file 1**. Data collection sheets for intravascular ultrasound image-guided liver navigation in an ex vivo model. The number of landmarks were 21 in the ex vivo model.

**Additional file 2**. Data collection sheets for intravascular ultrasound image-guided liver navigation in an in vivo model. The number of landmarks was 26 in the in vivo model. It included duodenum, pancreas, hepatic artery, and its left and right branches.

## Data Availability

The datasets generated and/or analyzed during the current study are available from the corresponding author on reasonable request.

## Abbreviations

IVUSc: Intravascular ultrasound catheter
US: Ultrasound
TIPS: Transjugular intrahepatic portosystemic shunts
DIPS: Direct intrahepatic portocaval shunts
IVC: Inferior vena cava
HV: Hepatic vein
PV: Portal vein
RJV: Right jugular vein
HA: Hepatic artery
