# Supplementary figures and images for: Fluoroless intravascular ultrasound image-guided liver navigation in porcine models
Source: BMC Gastroenterol. 2021 Jan 9;21:24. doi: 10.1186/s12876-021-01600-3 (PMC7797115; doi:10.1186/s12876-021-01600-3)

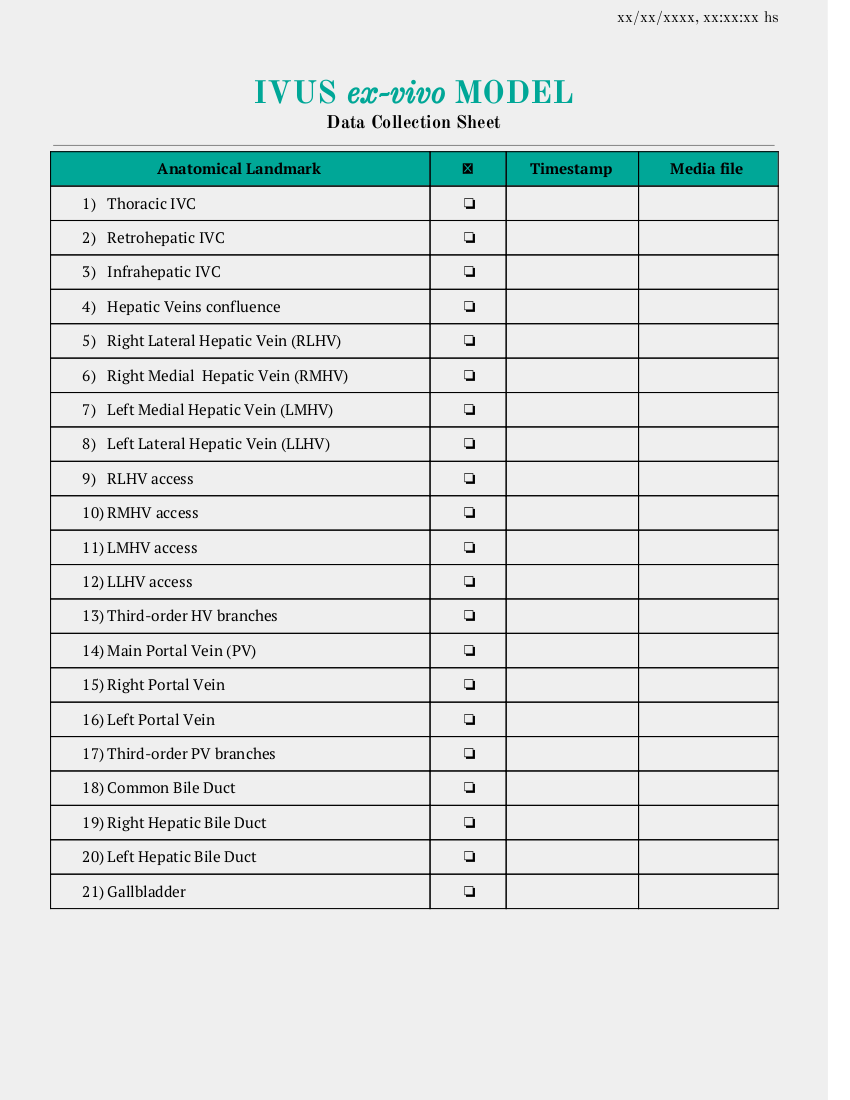

Supplement: Supplementary file 1 — Additional file 1. Data collection sheets for intravascular ultrasound image-guided liver navigation in an ex vivo model. The number of landmarks were 21 in the ex vivo model. [file 12876_2021_1600_MOESM1_ESM.tif]

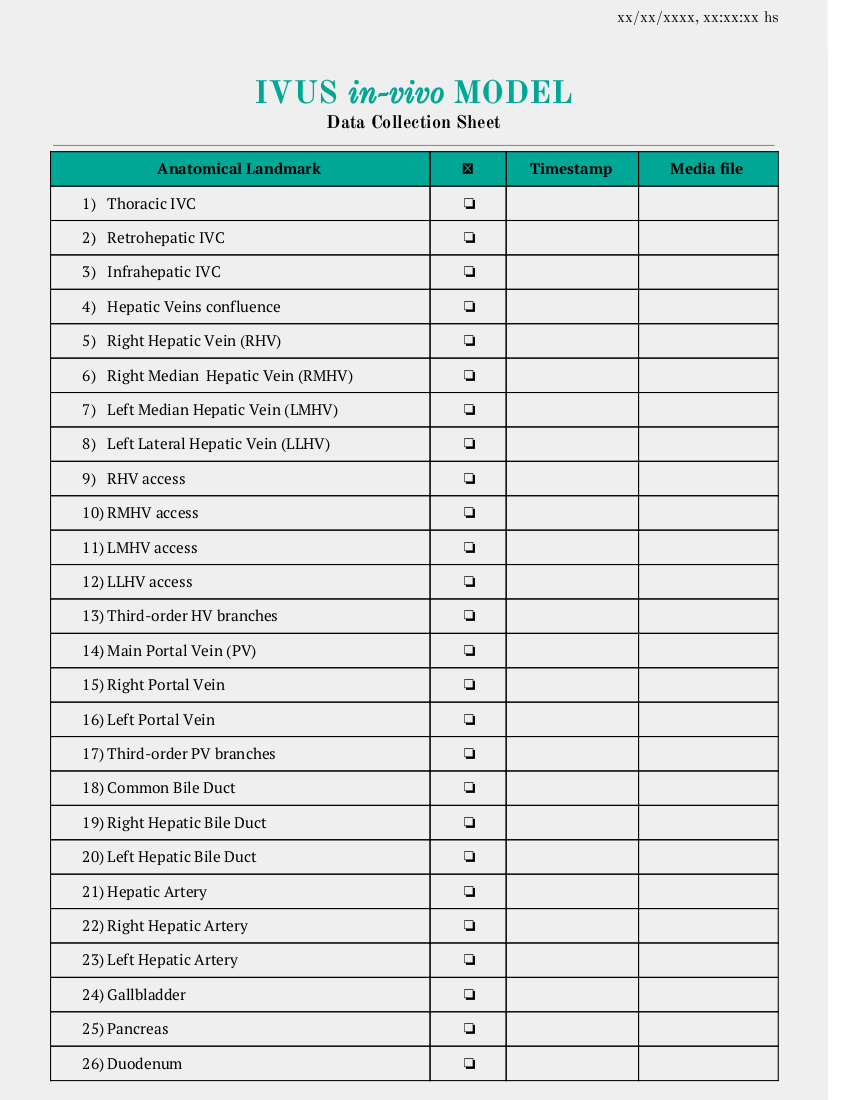

Supplement: Supplementary file 2 — Additional file 2. Data collection sheets for intravascular ultrasound image-guided liver navigation in an in vivo model. The number of landmarks was 26 in the in vivo model. It included duodenum, pancreas, hepatic artery, and its left and right branches. [file 12876_2021_1600_MOESM2_ESM.tif]
